# Asbestos Exposure in Patients with Malignant Pleural Mesothelioma included in the PRIMATE Study, Lombardy, Italy

**DOI:** 10.3390/ijerph19063390

**Published:** 2022-03-13

**Authors:** Andrea Spinazzè, Dario Consonni, Francesca Borghi, Sabrina Rovelli, Andrea Cattaneo, Carolina Zellino, Barbara Dallari, Angela Cecilia Pesatori, Hans Kromhout, Susan Peters, Luciano Riboldi, Domenico Maria Cavallo, Carolina Mensi

**Affiliations:** 1 Department of Science and High Technology, University of Insubria, 22100 Como, Italy; francesca.borghi@uninsubria.it (F.B.); sabrina.rovelli@uninsubria.it (S.R.); andrea.cattaneo@uninsubria.it (A.C.); domenico.cavallo@uninsubria.it (D.M.C.); 2 Occupational Health Unit, Fondazione IRCCS Ca’ Granda Ospedale Maggiore Policlinico, 20122 Milan, Italy; dario.consonni@policlinico.mi.it (D.C.); carolina.zellino@policlinico.mi.it (C.Z.); barbara.dallari@policlinico.mi.it (B.D.); angela.pesatori@unimi.it (A.C.P.); luciano.riboldi@unimi.it (L.R.); carolina.mensi@unimi.it (C.M.); 3 Department of Clinical Sciences and Community Health, University of Milan, 20122 Milan, Italy; 4 Institute for Risk Assessment Sciences, Utrecht University, 3584 CM Utrecht, The Netherlands; h.kromhout@uu.nl (H.K.); s.peters@uu.nl (S.P.)

**Keywords:** mesothelioma, asbestos, occupational exposure, retrospective exposure assessment, job-exposure matrix (JEM), SYN-JEM

## Abstract

The PRIMATE study is an Italian translational research project, which aims to identify personalized biomarkers associated with clinical characteristics of malignant pleural mesothelioma (MPM). For this purpose, characteristics of MPM patients with different degrees of asbestos exposure will be compared to identify somatic mutations, germline polymorphism, and blood inflammatory biomarkers. In this framework, we assessed exposure to asbestos for 562 cases of MPM extracted from the Lombardy region Mesothelioma Registry (RML), for which a complete interview based on a standardized national questionnaire and histopathological specimens were available. Exposure assessment was performed: (1) through experts' evaluation (considered as the gold standard for the purpose of this study), according to the guidelines of the Italian National Mesothelioma Registry (ReNaM) and (2) using a job-exposure matrix (SYN-JEM) to obtain qualitative (ever/never) and quantitative estimates of occupational asbestos exposure (cumulative exposure expressed in fibers per mL (f/mL)). The performance of SYN-JEM was evaluated against the experts' evaluation. According to experts' evaluation, occupational asbestos exposure was recognized in 73.6% of men and 23.6% of women; furthermore, 29 men (7.8%) and 70 women (36.9%) had non-occupational exposure to asbestos. When applying SYN-JEM, 225 men (60.5%) and 25 women (13.2%) were classified as occupationally exposed, with a median cumulative exposure higher for men (1.7 f/mL-years) than for women (1.2 f/mL-years). The concordance between the two methods (Cohen’s kappa) for occupational exposure assessment was 0.46 overall (0.41 in men, and 0.07 in women). Sensitivity was higher in men (0.73) than in women (0.18), while specificity was higher in women (0.88) than in men (0.74). Overall, both methods can be used to reconstruct past occupational exposure to asbestos, each with its own advantages and limitations.

## 1. Introduction

The term “Asbestos” refers to a group of silicate minerals classified into two mineralogical groups: serpentine mineral (i.e., chrysotile) and amphiboles (i.e., amosite, crocidolite, anthophyllite, tremolite, and actinolite) [1]. Asbestos fibers were widely used in the 20th century in many industrial sectors because of their characteristics (e.g., fire resistance, conductivity, thermal and noise insulation capacity, mechanical resistance, etc.). The highest asbestos exposure is generally expected in occupational environments, where manipulation processes may release asbestos fibers and expose workers [2]. All types of asbestos are classified as carcinogenic [3], and personal exposure to all types of asbestos increases the risk of developing asbestos-related diseases (ARDs) [3,4]. The typical ARDs can be divided into two categories: (i) non-malignant, including pleural plaques and asbestosis, and (ii) malignant, including lung, ovary, and larynx cancer and mesothelioma (all sites). The main risk factor for malignant mesothelioma (MM) is asbestos [5]. A high impact of asbestos exposure on malignant pleural mesothelioma (MPM) and peritoneal malignant mesothelioma occurrence has been shown in the Lombardy region, northwest Italy [6,7,8,9,10]. The incidence of MM has been rising in Lombardy in the last two decades, and the magnitude of the increase (+3.6% and +3.3% cases per year in men and women, respectively) is such that it cannot simply be attributed to increased diagnostic attention [6]. Recent studies also confirmed positive associations of asbestos with pericardial and tunica vaginalis testis mesothelioma [11,12,13]. 

Retrospective exposure assessment is one of the key elements in the identification of subjects exposed to asbestos and to examine the association between asbestos exposure and ARDs occurrence in epidemiological studies. This is due that (i) ARDs are characterized by a long latency (e.g., in the order of decades for MM) and because (ii) nowadays, several countries have banned or severely regulated asbestos (i.e., the ban from the Italian market dates back to 1992) [14,15]. Therefore, in these countries, except for operators still nowadays involved in asbestos removal (due to the widespread historical use of asbestos, there are still substantial quantities of this material in commercial, public and residential buildings), the scenarios of occupational exposure to asbestos fibers are no longer present and retrospective exposure assessment a necessity [2,16,17].

This study aims to conduct a retrospective exposure assessment for a group of residents in Lombardy who were diagnosed with MPM and enrolled in the "PRIMATE" project (“An integrated precision medicine approach to malignant mesothelioma: from mutation load to epidemiology and therapy”), within which this study was conducted. The PRIMATE study is a translational research project that aims to identify personalized biomarkers associated with clinical characteristics of the MPM. For this purpose, characteristics of patients with different degrees of asbestos exposure will be compared to identify (i) somatic mutations, (ii) germline polymorphisms, and (iii) blood inflammatory biomarkers (i.e., cytokines and metabolite). In the present work, lifetime asbestos exposure has been assessed with two methods: (i) using experts' evaluations to evaluate occupational and non-occupational exposure qualitatively, and then (ii) using a quantitative job-exposure matrix for studies of the general population (SYN-JEM), to obtain qualitative (ever/never) and quantitative estimates of cumulative occupational exposure. Results of the two methods were compared (only for occupational exposure), taking experts' evaluation as the gold standard.

## 2. Materials and Methods

### 2.1. Malignant Pleural Mesothelioma Patients

The study sample was selected from the Lombardy Mesothelioma Registry (“Registro Mesoteliomi Lombardia”, RML) in the period 2000–2019. As described by Mensi and collaborators [6], RML collects clinical information on all diagnosed malignant mesothelioma cases among Lombardy residents from regional and extra-regional hospitals. Since its implementation, diagnosis has been evaluated individually after revising clinical records by four experts working in the registry (CM, BD, CZ, and LR). Confirmed cases are classified as “definite” (histological diagnosis, possibly with immunohistochemical confirmation), “probable” (usually, cytology plus imaging), or “possible” (positive imaging), following guidelines of the Italian National Mesothelioma Registry (“Registro Nazionale Mesoteliomi”, ReNaM) [18]. Completeness of reporting (mandatory by the Italian law decree of the President of the Council of Ministers n. 308 of 10 December 2002) is periodically verified using various sources, including hospital admission databases and mortality registry. From the database, cases were selected among those (i) with available histopathological specimens in the pathology departments of four hospitals that participated in the PRIMATE study and for which (ii) a complete interview (i.e., standardized ReNaM questionnaire) was available. These were requirements defined upstream in the PRIMATE project, within which this study was conducted. 

### 2.2. Retrospective Assessment of Exposure to Asbestos

As previously stated, two methods of retrospective exposure assessment were applied in this study. The first method, to be considered as the reference method for the present study, relies on the RML evaluation of exposure. Subjects with confirmed MPM or their next-of-kin were interviewed by trained personnel using a standardized questionnaire covering lifetime job history (including industry, occupation, and details about tasks or indirect exposure within each job), as well as other various sources of extra-occupational asbestos exposure, including familial or para-occupational exposure (e.g., from a cohabitant's contaminated clothes), domestic or home-related exposure (e.g., ironing on asbestos boards; installation, repair or removal of asbestos-containing materials), and environmental exposure (mostly residence near an asbestos-cement factory) [6,7,18]. Then, following ReNaM guidelines [18], lifetime asbestos exposure was finally classified as “occupational” (definite, probable, or possible), “para-occupational” (i.e., related to the cohabitants), “home-related” (i.e., related to activities performed within the house), or “environmental" (residence in the vicinity of asbestos industries). For subjects with several exposure sources, asbestos exposure was classified according to this hierarchy: occupational > para-occupational > environmental > home-related. 

The second method was based on the linkage of the job history to SYN-JEM [19,20]. Coding of industries and jobs was performed by a unique person (CM) using the ATECO-91 Italian ISTAT-CIP-91 coding systems, respectively [21,22]. Consequently, a cross-walk tool [23] was applied to translate ISTAT-CIP-91 codes into ISCO-1968 codes [24]. Then, subjects' ISCO-68 codes were linked to SYN-JEM to get yearly intensities of exposure in airborne fibers per mL (f/mL) for each job. Cumulative occupational asbestos exposure (f/mL-years) was calculated by summing yearly intensities over individual job histories.

### 2.3. Statistical Analysis

Occupational exposure results (ever/never exposed) of the two methods were compared, taking experts' evaluation as the gold standard. We first calculated JEM sensitivity and specificity. Then we calculated Cohen's kappa coefficient of agreement. Confidence intervals (CI) were calculated with the Agresti–Coull formula. We additionally stratified analyses by gender. Data management and statistical analyses were performed with Stata 17 (StataCorp. 2021. Stata Statistical Software: Release 17. StataCorp LLC, College Station, TX, USA). Qualitative non-occupational exposure was also investigated using experts evaluations, as described above: these results have not been subjected to statistical analysis but are presented in the text to complete the information about the subjects’ exposure.

## 3. Results

In the 2000–2019 period, out of more than 7000 MPM cases in the RML database, we selected 562 subjects (372 men, 190 women) with characteristics eligible for this study (Table 1; Section 2.1). The mean age at diagnosis was about 69 years for both men and women. About 30% of cases were diagnosed in 2000–2009. Most (>98%) were definite diagnoses (only 8 cases were classified as probable MPM despite histological exam), and the most frequent (>70%) histological type was epithelioid. Pleural plaques were recognized in 17.5% and 14.7% of men and women, respectively, while asbestosis was recognized only in 2.1% of men. The proportion of cases personally interviewed was 75% and 69% for men and women, respectively. The average latency time (i.e., the time between the first attributed exposure and the diagnosis) was 48.8 years for men and 52.5 years for women, or 48.1 (men) and 53.3 (women) years if considering the first attributed occupational exposure. 

According to exposure evaluation by experts based on the ReNaM guideline, in men, definite occupational exposure was recognized in most cases (66.4%), while probable (0.8%) or possible (6.4%) occupational exposures were less frequent. In women, occupational exposure (18.4% definite, 1.0% probable, and 4.2% possible) was less frequently recognized than in men. For a non-negligible proportion of subjects (18.5% in men, 39.5% in women), no occupational exposure to asbestos was identified. Para-occupational, home-related, and environmental asbestos exposures were more frequently recognized in women (9.5%, 5.3%, and 22.1%, respectively) than in men (1.9%, 0.5%, and 5.4%, respectively). 

Concerning subjects' job histories, based on the collected information, exposed men were mostly employed in metalworking and metallurgy (33.6%), construction (26.6%), and asbestos-cement (11.7%) industry, with a mean duration of occupational exposure of 23.6 years. Exposed women were mostly employed in the non-asbestos-textile and clothing production (48.9%), health and social services (17.8), metalworking, and metallurgy sector (11.1%), with a mean duration of occupational exposure of 16.1 years.

**Figure 1:**
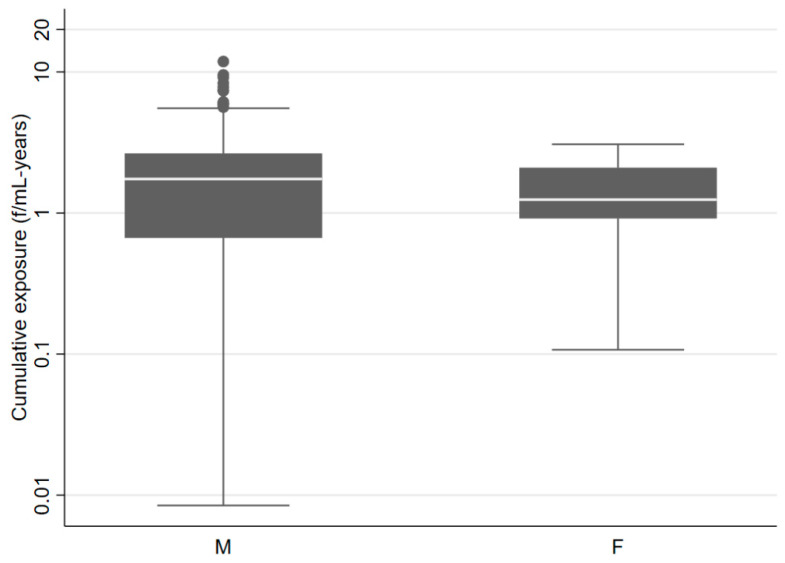
Cumulative exposure to asbestos (f/mL-years) estimated through the application of the SYN-JEM male (M) and female (F) subjects included in the PRIMATE study, Lombardy, Italy, 2000–2019 (only results for subjects occupationally exposed by JEM were plotted).

When applying SYN-JEM, 154 men (41.4%) and 16 women (8.4%) were assigned occupational exposure to asbestos in, at least one period during their job history, with a median exposure time of 24.5 and 11.4 years for men and women, respectively. The median estimated cumulative exposure was found to be slightly higher for men (1.7 f/mL-years) than for women (1.2 f/mL-years). The range in cumulative exposure for men was considerably larger with higher maximum values (Figure 1).

When comparing the application of SYN-JEM with the expert-based evaluation (RML), we found a sensitivity of 0.65 and a specificity of 0.83 (Table 2). Sensitivity was higher in men (0.73) than in women (0.18), while specificity was higher in women (0.88) than in men (0.75). More in detail, 42 subjects (25 men, 17 women) were categorized as occupationally exposed to asbestos when applying the SYM-JEM but not following RML criteria. Conversely, 111 subjects (74 men, 37 women) were classified as occupationally exposed according to RML criteria but not occupationally exposed when applying the SYM-JEM. The agreement was 0.46 overall, 0.41 in men, and 0.07 in women. Notably, in subjects classified as exposed by both approaches, 45 (21.6%) had pleural plaques, which are markers of asbestos exposure.

## 4. Discussion

In patients affected with MPM, applying two methods of retrospective exposure assessment, we found that the majority of men and a minority of women had ever been occupationally exposed to asbestos. Occupational exposure (definite, probable, or possible) to asbestos was recognized by RML in 73.6% of men and 23.6% of women. When applying SYN-JEM, the exposed proportion was lower in both cases (60.5% in men and 13.2% in women). The JEM showed good sensitivity in men and good specificity in both genders. The apparently low estimated median cumulative exposure can be explained by the fact that only a minority of subjects had been employed in jobs in high exposure industries (e.g., asbestos-cement and railroad production and maintenance). In addition, using experts’ assessment, we found that a further 17% had asbestos exposure out of work setting.

### 4.1. Subjects Classified as Occupationally Exposed by Experts and Non-Exposed by JEM

We found 111 subjects (19.7%) classified as occupationally exposed according to experts but non-exposed when applying the SYM-JEM. Of these, 24 subjects were enrolled with different job tasks in the production of non-asbestos textile and cloth (one further subject was employed in this sector, as machine production employee) and were considered occupationally exposed to asbestos since they used sewing-machines or pressing machines [25,26,27]. Six subjects were involved as bakers or similar jobs and were considered occupationally exposed because asbestos-containing materials (ACMs) were recognized in ovens for baking bread manufactured prior to the 1980s [28]. Five subjects were employed as hairdressers: in these cases, RML staff already hypothesized and confirmed the existence of an occupational asbestos exposure risk (i.e., airborne asbestos fiber exposure generated by the use of hand-held or hood-type hairdryers containing asbestos), at least in the past [29]. Two subjects were employed as a machine packer and a rotary pressman in a newspaper printing industry; for this job, RML already recognized and discussed an occupational asbestos exposure risk (i.e., asbestos used for insulation of the building structure, rather than and not of the working equipment) [30]. Ten subjects were employed (but not as production workers) in asbestos-cement production, which, moreover, belongs to a cluster of subjects whose occupational exposure was already described in a previous study [10]. Eight subjects worked as office employees in different industrial sectors (i.e., asbestos texture, rubber production): in these cases, it was recognized (during the RML interview) that to carry out their task, these subjects went daily to production departments, in which the presence of insulation on building structures and/or operating systems had been recognized [31,32]. Other subjects worked with different job titles in different sectors (e.g., steel mills and pipe manufacturing, metalworking or to produce machines or to produce and install plants, rubber and plastic production, processing of non-metallic minerals, etc.) for which they reported employment in departments with the presence and use of asbestos. Overall in these cases, although the jobs did not involve the direct use of asbestos, these workers could be classified as bystanders and thus occupationally exposed [33,34]. Two subjects who worked in the agricultural sector and were classified as occupationally exposed due to the existence of multiple sources of exposure to asbestos in the agricultural setting were defined [35]. For several other workers, evidence was collected regarding the occurrence of episodes of handling materials containing asbestos and opportunities for exposure to asbestos fibers in their workplace. As a last note, 19 out of 111 (17%) had pleural plaques.

### 4.2. Subjects Classified as Occupationally Exposed by JEM and Non-Exposed by Experts

We found 42 (7.5%) cases classified as occupationally exposed when applying the SYM-JEM but non-exposed, according to experts. We found five subjects with pleural plaques, but all were non-occupationally exposed, according to the experts. It has been previously advocated that the expert assessment to assign exposures provides more accurate exposure estimates than JEM-based results [36,37]. In a subsequent study, Peters and colleagues [38] have compared the results of different exposure assessment methodologies in a case-control study: results outlined poor agreement between experts assessments and results of a general population JEM (i.e., DOM-JEM) for asbestos (k = 0.17). A better agreement (k = 0.53) was found between experts assessments and a population-specific JEM (PSJEM) based on the results of expert assessment within the study [38]. In the present study, the agreement was 0.46 overall (0.41 in men and 0.07 in women), which is in the same range as the inter-rater agreement found in previous studies [38,39]. It was therefore argued that the accuracy of exposure assessment for asbestos is based more on specific circumstances than on job title alone, and more in general that the reliability of the assessment also depends, to some extent, on the type of exposure of interest [38]. In fact, in the present study, experts assessment relied on detailed information collected at interview through a standardized questionnaire on occupational history, which includes industries and jobs, and specific tasks and information on workplaces [18]. Other sources of information are also employed, including notification by the local health authority about the presence of asbestos in the work environment, the presence of other ARDs in the same company, and their description of working conditions. 

### 4.3. Advantages and Limitations of the Two Approaches

Then, it is evident that both the adopted methods have their own advantages and disadvantages that need to be considered when assessing the exposure and analyzing the outcomes. For example, the experts’ evaluation is based on information that can be affected by recall, interviewer, and assessor biases, which could result in a biased exposure assessment. However, experts’ evaluation could explore the patient’s occupational and extra-occupational history and allow greater detail in the characterization of the subjects and evaluate further information. In summary, although it can be considered detailed and robust (the evaluation RML is only qualitative, while SYN-JEM also provides quantitative data) and can undergo subjectivity. 

In this regard, the SYN-JEM has some main advantages over expert assessment: (i) it is much less time-consuming; (ii) it results in quantitative exposure estimates, and (iii) it is not affected by recall and interviewer biases as it is based only on job titles (the quality of job coding and crosswalks do play an important role, anyway).

On the other hand, when applying the SYN-JEM, there may be an underestimation due to non-differential exposure misclassification because of lower accuracy and possible errors in coding occupations. About this last point, coding of job titles from each subject's job history is often challenging and could result in misclassification of exposure [40,41,42,43,44,45]. Anyhow, the method applied in this study showed good performances (i.e., sensitivity, specificity, agreement with manual coding) and, overall, it was considered reliable and applicable to efficiently translate Italian job codes (ISTAT-CIP-91) to international job codes (ISCO-68) for the subsequent application of JEMs [23]. 

In this regard, the high level of standardization and ease of use represent an advantage and make JEMs useful for large-scale studies while at the same time representing drawbacks [40,46]. More in detail, JEMs generally fail to characterize the inter-individual variability and heterogeneity of exposure of different workers classified in the same job title or temporal variations in exposure levels [40]. However, given a group-based approach and the associated Berkson-type error, it will result in no or little bias of risk estimates but with loss of precision [47]. On the other hand, the use of JEM alone allows to adopt an objective method, but which is not able to assess non-occupational exposure and which is not able to adequately assess occupational exposure heterogeneity, within-job (i.e., between-worker) variability, temporal (i.e., within-worker) variability and long-term trends in exposure [16,40]. Anyhow, it should be noted that results of a large multicenter study outlined when using the results of experts assessments and JEM in epidemiological analysis, both methods gave similar results as to whether occupational exposure to asbestos increases lung cancer risk. Thus, it was argued that the advantages of experts assessment, when compared to a JEM approach, could be little (the DOM-JEM resulted in being faster and cheaper anyway). However, an agreement between methods can depend on several factors, including (but not limited to) (i) the skill and experience of the involved experts; (ii) the time invested in the entire process of expert assessment, and (iii) the quality and number of documentary sources available [38]. Thus, overall, it remains unknown to what extent these general insights could be generalized to other studies, and a case-by-case evaluation should be made. 

In summary, there is no single approach capable of providing an accurate and comprehensive estimate of exposure considering all necessary contextual information; thus, the best approach to be adopted depends on the level of information available for the specific case and the required level of detail for results. Generally, using a combination of different REA techniques can represent a solution for weighting and integrating data obtained through qualitative and quantitative approaches to obtain the best possible estimate [16]. For this reason, although expert evaluation is considered the reference method for this study, both RML and SYN-JEM results will be used in PRIMATE. This will be essential to get the most complete possible picture of asbestos exposure in the subjects recruited for the project and to classify the subjects included in the study accordingly. Therefore, the authors reiterate that the combination of clinical and technical-scientific skills in occupational and environmental hygiene and exposure sciences is of fundamental importance for the optimal characterization of exposure. It is evident that the proposed classification provides, in any case, a certain degree of simplification and that several other variables associated with each subject must be necessarily considered in addition to this classification to continue in more in-depth studies in the framework of the PRIMATE project. The main limitation refers to the impossibility of obtaining a quantitative evaluation of the cumulative occupational exposure of some subjects (i.e., those considered not occupationally exposed by using SYN-JEM) and the impossibility of quantifying non-occupational exposures (i.e., “home-related” and “environmental” exposure) for all subjects, which were qualitatively classified based on the RML assessment, anyway.

## 5. Conclusions

Retrospective exposure assessment for 562 cases of MPM included in the PRIMATE study was performed through experts' evaluation (considered as the gold standard) and by applying SYN-JEM. As expected, the findings obtained from the two methods were not completely consistent. Overall, weighing the strengths and drawbacks of each method, using a combination of different retrospective exposure assessment techniques represented the solution to obtain the best possible estimate for the study at hand.

The choice between the two approaches depends in part on the objectives of the exposure assessment. The expert method is, in general, to be preferred when assessing exposure in medico-legal settings (e.g., for compensation purposes), while the JEM method is most suitable for epidemiological studies.

## Figures and Tables

**Table 1:** Characteristics of 562 subjects with malignant pleural mesothelioma (MPM) and interview selected for the PRIMATE study, Lombardy, Italy, 2000–2019.

	Men		Women	
	N	%	N	%
Total	372	100	190	100
**Age at diagnosis (years) mean (min-max)**	68.8	(24.5–94.5)	69.3	(41.3–93.1)
**Period of diagnosis**				
2000–2009	111	29.8	64	33.7
2010–2019	261	70.2	126	66.3
**Diagnosis**				
Definite	367	98.7	187	98.4
Probable	5	1.3	3	1.6
**Morphology (ICD-O code) ^#^**				
MM not otherwise specified (90503)	6	1.6	5	2.6
Fibrous/sarcomatoid/desmoplastic MM (90513)	20	5.4	15	7.9
Epithelioid MM (90523)	262	70.4	148	77.9
Biphasic MM (90533)	84	22.1	22	11.6
**Presence of pleural plaques**	65	17.5	28	14.7
**Presence of asbestosis**	8	2.1	0	0.0
**Interview**				
Patient	279	75.0	131	69.0
Relative	93	25.0	59	31.0
**Vital status**				
Dead	352	94.6	177	92.7
Alive	20	5.4	13	6.8
**Asbestos exposure (ReNaM)**				
Occupational, definite	247	66.4	35	18.4
Occupational, probable	3	0.8	2	1.0
Occupational, possible	24	6.4	8	4.2
Para-occupational	7	1.9	18	9.5
Home-related	2	0.5	10	5.3
Environmental	20	5.4	42	22.1
None identified	69	18.5	75	39.5
**Years since first asbestos exposure (RML)** **mean (min–max)**	48.8	(18.5–78.1)	52.5	(16.6–85.1)
**Years since first occupational asbestos exposure (RML)** **mean (min–max)**	48.1	(18.5–71.5)	53.3	(17.8–69.6)
**Duration of occupational asbestos exposure (RML)** **mean (min–max)**	23.6	(0.5–60.4)	16.1	(1.0–43.0)
Occupational asbestos exposure: industry * (RML)				
Metalworking and metallurgy	92	33.6	5	11.1
Construction	73	26.6	0	0.0
Asbestos-cement	32	11.7	2	4.4
Textile and clothing production	8	2.9	22	48.9
Motor vehicle production	20	7.3	0	0.0
Transport	16	5.8	0	0.0
Chemical	15	5.5	0	0.0
Railroad production and maintenance	15	5.5	0	0.0
Food and beverage	11	4.0	2	4.4
Health and social services	4	1.5	8	17.8
Military	10	3.6	0	0.0
**Occupational asbestos exposure (SYN-JEM)**				
Ever	225	60.5	25	13.2
Cumulative (f/mL-years) median (min–max)	1.7	(0.01–11.9)	1.2	(0.1–3.1)
Duration (years) median (min–max)	24.5	(1–61)	11.4	(2.0–28.0)

^#^ ICD-O, International Classification of Diseases for Oncology, Third Edition; MM, malignant mesothelioma. * Only industries with at least 10 exposed cases are listed; a subject may have been exposed to asbestos in more than one industrial sector in his/her occupational history.

**Table 2:** Comparison of occupational asbestos exposure from the job-exposure matrix (SYN-JEM) with expert-based assessment (RML) based on the national mesothelioma registry (ReNaM) guideline, PRIMATE study, Lombardy, Italy, 2000–2019.

Occupational	Men		Women		All	
Asbestos Exposure	RML		RML		RML	
	Ever	Never	Ever	Never	Ever	Never
SYN-JEM						
Ever	200	25	8	17	208	42
Never	74	73	37	128	111	201
Sensitivity	0.73		0.18		0.65	
95% CI *	0.67; 0.78		0.09; 0.32		0.60; 0.70	
Specificity		0.75		0.88		0.83
95% CI *		0.65; 0.82		0.82; 0.93		0.77; 0.87
Cohen’s kappa	0.41		0.07		0.46	
95% CI	0.34; 0.48		−0.01; 0.15		0.39; 0.53	

Abbreviations: CI, confidence interval. * Calculated with the Agresti–Coull formula.

## Data Availability

Not applicable.
